# Rupture of the Heart

**Published:** 1848-12

**Authors:** 


*Rupture of the Heart.*—A man, sixty-four years of age, whose heart was lately shown to the Academy of Medicine in Paris, was admitted into the hospital of Beaujon, Paris, on the 31st of August, under M. Bouvier, for pains in the epigastrium, loss of strength and appetite. Respiration was pretty free, but for two nights, which he spent in the hospital, he had hardly ever assumed the recumbent posture. On the 2d of September the pain occupied the left side of the chest, extending up to the left shoulder; it was, however, far from distressing, and being accompanied by no fever or dulness on percussion, it was set down as arising from rheumatism. In the afternoon, the patient complained all at once of severe headache and of great heat; he threw the windows wide open, and in a moment afterwards he was found on his bed, the head thrown back, the legs hanging, and the face quite purple. He was dead before any assistance could be rendered. On a post-mortem examination forty-two hours after death, the pericardium was found distended with blood; it contained about three ounces of a soft and dark coagulum, surrounding the heart, particularly posteriorly on the right side. The blood had escaped by a longitudinal fissure, situated on the anterior aspect of the right ventricle; this aperture is one inch and a third in length; the margins, rather irregular, are separated to the extent of four or five lines, the interval being filled up by a coagulum of recent formation, slightly adherent, and extending into the interior of the ventricle. The parietes of the latter are considerably thinned, and present at the lower angle a small aperture, which leads into the ventricle. The fissure, viewed internally, presents, at a little distance from it, a prominence formed by the internal muscular layers; the sides of the fissure itself are formed by a very thin stratum of the most external layers. The whole looks like an excavation scooped from the muscular substance, the thin fundus of which is marked by the rent which gave issue to the blood. The heart is of a normal size, the parietes are of the ordinary consistence, the various orifices are in a perfect state, with the exception of one of the aortic valves, which is ossified. The defective history of the case precludes the possibility of accurately determining the cause of this lesion; it is, however, likely that there was first a solution of continuity, with stretching, or even loss of substance of the carnæ columnæ, and that this state of things lasted some time before the blood broke down the thin external layer. The symptoms manifested by this patient have a certain analogy with those sometimes evinced by functional disturbance of the organ. Thus Morgagni mentions a case, (Epist. 27, No. 8,) where the patient had pains in the sternum and arms, and complained, in his last moments, of giddiness and tightening of the chest.—*London Lancet.*

